# Prenatal Lead Exposure, Genetic Factors, and Cognitive Developmental Delay

**DOI:** 10.1001/jamanetworkopen.2023.39108

**Published:** 2023-10-23

**Authors:** Zhenxian Jia, Hongling Zhang, Ling Yu, Feng Qiu, Yiqing Lv, Jing Guan, Huiqing Gang, Jingwen Zuo, Tongzhang Zheng, Hongxiu Liu, Wei Xia, Shunqing Xu, Yuanyuan Li

**Affiliations:** 1 Key Laboratory of Environment and Health, Ministry of Education and Ministry of Environmental Protection, and State Key Laboratory of Environmental Health (Incubation), School of Public Health, Tongji Medical College, Huazhong University of Science and Technology, Wuhan, Hubei, China; 2 Wuchang University of Technology, Wuhan, Hubei, China; 3 Department of Epidemiology, School of Public Health, Brown University, Providence, Rhode Island

## Abstract

**Importance:**

Although the effects of lead (Pb) exposure on neurocognition in children have been confirmed, the individual associations of prenatal Pb exposure and its interaction with genetic factors on cognitive developmental delay (CDD) in children remain unclear.

**Objective:**

To investigate the association of prenatal Pb exposure and its interaction with genetic factors with CDD risk.

**Design, Setting, and Participants:**

Women in Wuhan, China, who had an expected delivery date between March 2014 and December 2017, were recruited for this prospective cohort study. Children were assessed for cognitive development at approximately 2 years of age (March 2016 to December 2019). Maternal venous blood, cord blood, and venous blood from children were collected in a longitudinal follow-up. Data analysis was performed from March 2022 to February 2023.

**Exposure:**

Prenatal Pb exposure, and genetic risk for cognitive ability evaluated by polygenic risk score constructed with 58 genetic variations.

**Main Outcomes and Measures:**

Cognitive developmental delay of children aged approximately 2 years was assessed using the Chinese revision of the Bayley Scale of Infant Development. A series of multivariable logistic regressions was estimated to determine associations between prenatal Pb exposure and CDD among children with various genetic backgrounds, adjusting for confounding variables.

**Results:**

This analysis included 2361 eligible mother-child pairs (1240 boys [52.5%] and 1121 girls [47.5%]; mean [SD] ages of mothers and children, 28.9 [3.6] years and 24.8 [1.0] months, respectively), with 292 children (12.4%) having CDD. Higher maternal Pb levels were significantly associated with increased risk of CDD (highest vs lowest tertile: odds ratio, 1.55; 95% CI, 1.13-2.13), adjusting for demographic confounders. The association of CDD with maternal Pb levels was more evident among children with higher genetic risk (highest vs lowest tertile: odds ratio, 2.59; 95% CI, 1.48-4.55), adjusting for demographic confounders.

**Conclusions and Relevance:**

In this cohort study, prenatal Pb exposure was associated with an increased risk of CDD in children, especially in those with a high genetic risk. These findings suggest that prenatal Pb exposure and genetic background may jointly contribute to an increased risk of CDD for children and indicate the possibility for an integrated strategy to assess CDD risk and improve children’s cognitive ability.

## Key Points

**Question:**

Is prenatal lead (Pb) exposure together with genetic factors associated with the risk of cognitive developmental delay (CDD)?

**Findings:**

In this cohort study of 2361 mother-child pairs followed up prenatally to child age of approximately 2 years, prenatal exposure to even low levels of Pb was associated with an increased risk of CDD in children, especially those with a high genetic risk.

**Meaning:**

These findings suggest that prenatal Pb exposure and its interaction with genetic factors might jointly contribute to CDD risk, indicating the possibility of an integrated strategy for the assessment of CDD risk.

## Teaser

This cohort study investigates the association of prenatal lead exposure and its interaction with genetic factors with risk of cognitive development delay in children.

## Introduction

Lead (Pb) is an established toxic metal that impairs both children’s and adults’ health. Previous studies have shown that no detectable blood Pb levels are considered to be safe for children.^1,2,3^ Although blood Pb levels for both children and adults have been reduced substantially in recent years,^4,5,6^ emerging sources of Pb pollution are increasing.^7^ For some individuals, such as pregnant women, the mobilization of bone Pb stores during pregnancy also remains a concern.^8^

Converging evidence has shown that Pb readily passes through the placenta to reach the fetus and injure the fetus’s nervous system.^9^ In a study of 436 children from Poland, cord blood Pb levels were found to be negatively associated with fluid and crystallized IQ and mathematical skills.^10^ Conversely, a prospective birth cohort study from Japan, including 286 children, failed to find a significant association between prenatal Pb exposure and IQ.^11^ Currently, epidemiologic evidence regarding the association between prenatal Pb exposure and childhood cognition remains scarce. Cognitive developmental delay (CDD) in children due to prenatal Pb exposure is still not well assessed, and findings have been inconsistent.^12,13,14^

Genetic factors also contribute largely to the heterogeneity of an individual’s cognitive ability.^15,16,17^ Considering that complex phenotypes, such as cognition, usually involve a joint effect of multiple single nucleotide variants (SNVs) (formerly SNPs), a polygenic risk score (PRS) is proposed to increase the prediction of genetic susceptibility by weighing and integrating multiple single genetic variants with the lesser effect of cognition heritable risk.^18,19^ However, existing research has mainly concentrated on the separate association of Pb exposure or genetic variants with the risk of CDD, and their interplay is usually ignored. We conducted a prospective birth cohort study of mother-child pairs in China to investigate the hypothesis that prenatal Pb exposure and its interaction with genetic factors might jointly contribute to CDD risk and provide an integrated strategy for the assessment of CDD risk by bringing genetics and prenatal Pb exposure together.

## Methods

### Participants

This cohort study is part of the large, prospective Healthy Baby Cohort, which was conducted at the Wuhan Medical and Healthcare Center for Women and Children in Wuhan, China, with an aim to explore the associations of environmental, genetic, and other factors with child health development. Between March 2014 and December 2017, pregnant women who met the following criteria were recruited at their first antenatal visit: (1) resided in the study area (Wuhan, China); (2) were willing to undergo prenatal care and delivery at the research hospital; (3) had a single gestation and live birth; and (4) were willing to complete assessments and donate biological samples during regular visits. After recruitment, pregnant women were longitudinally followed in the first, second, and third trimesters of pregnancy. After childbirth, children’s regular follow-up assessments were carried out at specific time points, including 1 month, 6 months, 1 year, 2 years, 3 years, 5 years, 6 years, and so on. Between March 2016 and December 2019, when the children were aged approximately 2 years (mean [SD], 24.8 [1.0] months), they were followed up for a cognitive development assessment.

The human investigation committee of the Wuhan Medical and Healthcare Center for Women and Children and Tongji Medical College of Huazhong University of Science and Technology approved this work for research ethical purposes. Following a thorough description of the study’s objectives and methods, each mother provided signed informed consent. The informed consent for children was signed by a guardian. This report follows the Strengthening the Reporting of Observational Studies in Epidemiology (STROBE) reporting guideline for cohort studies.

### Evaluation of Cognitive Functions in Children

The cognitive development of children (mean [SD] age, 24.8 [1.0] months) was analyzed using a variation of the Bayley Scales of Infant Development,^20^ which was adapted for application in the Chinese population.^21,22^ As with its precursor, the Chinese revision of the Bayley Scales of Infant Development includes scales for the mental development index (MDI) and psychomotor development index. Specifically, the MDI is a representation of a child’s cognitive development with respect to language ability, generalization, categorization, memory, and social skills, among other aspects. An MDI score of less than or equal to 79 is considered indicative of CDD. Further details are presented in eMethods 1 in Supplement 1.

### Measurement of Exposure to Pb

To assess Pb exposure, maternal fasting venous blood was collected during the first trimester. Immediately after collection, low-speed centrifugation was used to isolate plasma from venous blood. After isolating the plasma, hemolysis was preliminarily assessed by observation (reddening), assaying for the presence of hemoglobin in the resulting plasma as a quality control step.^23,24^ The plasma was then placed at −80 °C for further measurement of Pb levels. An inductively coupled plasma mass spectrometer (Agilent 7900; Agilent Technologies) was used to measure Pb concentrations in the plasma. For children aged 2 years, venous blood samples were collected and placed at −80 °C. Subsequently, a Trace Element Analyzer AS-9000C (Wuhan Aoshou Medical Technology Co, Ltd) was used to measure the Pb concentrations of whole blood in accordance with standard operating procedures. The detailed protocols are presented in eMethods 2 in Supplement 1.

### PRS Calculation

To construct the PRS, the experimental cohort of children was screened for those whose DNA samples yielded successful genotyping assays and passed quality control (eMethods 3-5 in Supplement 1). Recently, the Cohorts for Heart and Aging Research in Genomic Epidemiology consortium conducted a meta-analysis of genome-wide association studies and identified 361 SNVs that are believed to have an association with cognitive function (*P* < 1 × 10^−5^).^25^ Of those SNVs, 325 were available in the imputed genotype data of the subject children after quality control. Subsequently, linkage disequilibrium clumping was performed to ensure independence among SNVs (*R*^2^ = 0.8, 500-kilobase window). After filtering, 58 retained SNVs with low linkage disequilibrium were used to construct the PRS for this study (eMethods 6 in Supplement 1). As described previously, the PRS tertiles were based on their distribution among all the children, the lowest of which was categorized as high genetic risk.^26,27^

### Statistical Analysis

The data analysis was performed from March 2022 through February 2023. Participants’ general characteristics are presented as counts and percentages for categorical variables or means with SDs for continuous variables. The distributions of Pb levels in mothers and children are presented as percentiles and geometric means (GMs) with geometric SDs. The multivariable logistic regression model was used to estimate the associations between prenatal Pb exposure and CDD risk. The covariates (eMethods 7 in Supplement 1) for children (sex), mothers (maternal age at delivery [continuous], prepregnancy body mass index[continuous], maternal education status [college or more, some college, high school or less], parity [1, ≥2], mode of delivery [vaginal, cesarean], gestational age at birth [>37 weeks, ≤37 weeks]), fathers (paternal education status [college or more, some college, high school or less]), family (annual family income [<50 000 yuan, 50 000-99 000 yuan, ≥100 000 yuan]), the top 10 genetic principal components (continuous) (when appropriate), and blood Pb levels in children (log-transformed) (when appropriate) were adjusted as potential confounding factors. To detect additive interactions, we used a method outlined by Andersson et al.^28^ For evaluating the additive interaction, we calculated the relative excess risk due to interaction (RERI) and the attributable proportion due to the interaction (AP). The CIs of RERI and AP do not include 0 if there is a significant additive interaction.

To test the robustness of the study findings, multiple sensitivity analyses were performed. First, we excluded children with abnormal birth weight (<2500 g or >4000 g) to avoid confounding effects of intrauterine dysplasia.^29,30^ Second, we excluded mothers with gestational diabetes and gestational hypertension to avoid confounding with neurocognitive development in children from common pregnancy complications.^31,32^ All *P* values were 2-sided. The significant *P* value was set at .05 and the suggestive significant *P* value (providing an initial indication or trend) at .20, according to the χ^2^ test, unpaired 2-sample *t* test, generalized linear model, and multivariable logistic regression model. All analyses were conducted using R, version 4.1.1 (R Foundation for Statistical Computing) and SAS, version 9.4 (SAS Institute Inc) statistical software.

## Results

**Figure 1.**
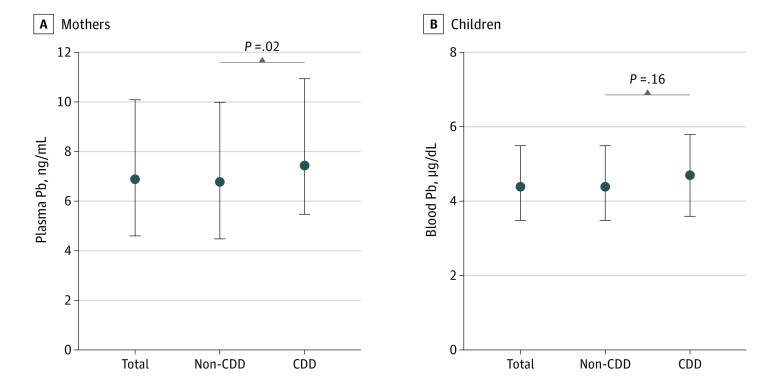
Distributions of Lead (Pb) Concentrations Among Mother-Child Pairs The geometric mean (geometric SD) of plasma Pb concentrations in mothers and blood Pb concentrations in children are 7.32 (0.12) ng/mL (to convert ng/mL to g/L, multiply by 1 × 10^−6^) and 4.39 (0.04) μg/dL (to convert μg/dL to g/L, multiply by 1 × 10^−5^), respectively. Circles and whiskers represent the median and IQR, respectively. CDD indicates cognitive developmental delay.

After excluding ineligible participants (eFigure 1 in Supplement 1), 2361 eligible mother-child pairs made up the analytic sample (mean [SD] age, 28.9 [3.6] years for mothers and 24.8 [1.0] months for children; 1240 boys [52.5%] and 1121 girls [47.5%]). Of these mother-child pairs, 292 children (12.4%) were found to have CDD at age approximately 2 years. Table 1 and eTable 1 in Supplement 1 show the distribution of demographic characteristics. The GM (geometric SD) of Pb concentrations measured in mothers’ plasma and children’s whole-blood samples were 7.32 (0.12) ng/mL and 4.39 (0.04) μg/dL, respectively (Figure 1; eTable 2 in Supplement 1). Child sex (girls vs boys: odds ratio [OR], 0.43; 95% CI, 0.33-0.56), mode of delivery (vaginal vs cesarean: OR, 1.33; 95% CI, 1.04-1.70), annual family income (<50 000 vs ≥100 000 yuan: OR, 1.70; 95% CI, 1.21-2.39), maternal education status (high school or less vs college or more: OR, 1.57; 95% CI, 1.16-2.14), and paternal education status (high school or less vs college or more: OR, 1.64; 95% CI, 1.22-2.20), were significantly associated with CDD (eFigure 2 in Supplement 1).

**Table 1.** Descriptive Characteristics of All Mother-Child Pairs

Characteristic	Mother-child pairs, No. (%)			*P* value^a^
	Total (N = 2361)	No CDD (n = 2069)	CDD (n = 292)	
Maternal age at delivery, mean (SD), y	28.9 (3.6)	29.0 (3.8)	28.8 (3.5)	.49
Maternal age group at delivery, y				
≤24	182 (7.7)	155 (7.5)	27 (9.2)	.20
25-29	1348 (57.1)	1198 (57.9)	150 (51.4)	
30-34	644 (27.3)	555 (26.8)	89 (30.5)	
≥35	187 (7.9)	161 (7.8)	26 (8.9)	
Prepregnancy BMI, mean (SD)	20.9 (2.9)	21.3 (3.1)	20.9 (2.8)	.04
Prepregnancy BMI group				
<18.5	473 (20.0)	420 (20.3)	53 (18.2)	.09
18.5-23.9	1557 (65.9)	1371 (66.3)	186 (63.7)	
≥24	331 (14)	278 (13.4)	53 (18.2)	
Annual family income, yuan/y				
≥100 000	1120 (47.4)	996 (48.1)	124 (42.5)	.009
50 000-99 000	914 (38.7)	803 (38.8)	111 (38)	
<50 000	327 (13.9)	270 (13.0)	57 (19.5)	
Maternal education status				
College or more	1190 (50.4)	1061 (51.3)	129 (44.2)	.01
Some college	698 (29.6)	611 (29.5)	87 (29.8)	
High school or less	473 (20)	397 (19.2)	76 (26)	
Paternal education status				
College or more	1039 (44)	926 (44.8)	113 (38.7)	.002
Some college	769 (32.6)	682 (33)	87 (29.8)	
High school or less	553 (23.4)	461 (22.3)	92 (31.5)	
Passive smoking during pregnancy				
No	1777 (75.3)	1551 (75.0)	226 (77.4)	.37
Yes	584 (24.7)	518 (25.0)	66 (22.6)	
Parity				
1	1961 (83.1)	1724 (83.3)	237 (81.2)	.36
≥2	400 (16.9)	345 (16.7)	55 (18.8)	
Mode of delivery				
Vaginal birth	1174 (49.7)	1047 (50.6)	127 (43.5)	.02
Cesarean birth	1187 (50.3)	1022 (49.4)	165 (56.5)	
Gestational age at birth, mean (SD), wk	39.0 (1.2)	39.0 (1.2)	39.0 (1.1)	.62
Gestational age group at birth, wk				
>37	2278 (96.5)	1993 (96.3)	285 (97.6)	.27
≤37	83 (3.5)	76 (3.7)	7 (2.4)	
Child sex				
Male	1240 (52.5)	1036 (50.1)	204 (69.9)	<.001
Female	1121 (47.5)	1033 (49.9)	88 (30.1)	

Abbreviations: BMI, body mass index (measured as weight in kilograms divided by height in meters squared); CDD, cognitive developmental delay.

^a^ *P* value for the difference according to the χ^2^ test and unpaired 2-sample *t* test.

Higher plasma Pb concentrations in pregnant women were associated with a significantly enhanced risk for CDD in children. In the unadjusted model (model 1), CDD risk was associated with increasing concentrations of Pb (highest vs lowest tertile: crude OR, 1.63; 95% CI, 1.20-2.22; *P* for trend = .002) (Table 2). After adjustment for demographic confounding factors (model 2), the positive association between CDD risk and prenatal plasma Pb levels was similarly elevated (highest vs lowest tertile: adjusted OR, 1.55; 95% CI, 1.13-2.13; *P* for trend = .01) (Table 2). We additionally adjusted the children’s blood Pb levels to exclude interference from childhood Pb exposure (model 3), and the association of prenatal Pb exposure with CDD risk yielded similar results (highest vs lowest tertile: adjusted OR, 1.98; 95% CI, 1.28-3.07; *P* for trend = .005) (Table 2; full regression analysis provided in eTable 10 in Supplement 1). Stratified analyses by sex showed that prenatal Pb exposure was more prominent for girls (highest vs lowest tertile, adjusted for demographic confounders: adjusted OR, 2.04; 95% CI, 1.16-3.58; *P* for trend = .008) (eTable 3 in Supplement 1). This study has also shown that the association between children’s CDD risk and Pb exposure at age approximately 2 years (eTable 4 in Supplement 1) was relatively lower compared with prenatal Pb exposure. In sensitivity analyses, we found that the results were appreciably unchanged after excluding children with abnormal birth weight (<2500 g or >4000 g) (eTable 5 in Supplement 1). The CDD risk for prenatal Pb exposure was also stable in the analysis excluding mothers with gestational diabetes or gestational hypertension (eTable 6 in Supplement 1). However, the analyses for motor developmental delay found that increased prenatal Pb exposure did significantly increase CDD risk (eTable 7 in Supplement 1).

**Table 2.** Associations Between Cognitive Developmental Delay (CDD) Risk and Prenatal Lead Exposure

Prenatal lead exposure^a^	No. of children		OR (95% CI)	*P* value	*P* value for trend^b^
	Total	CDD			
Model 1					
Lowest tertile	787	76	1 [Reference]	NA	.002
Intermediate tertile	786	99	1.35 (0.98-1.85)	.06	
Highest tertile	788	117	1.63 (1.20-2.22)	.002	
Model 2					
Lowest tertile	787	76	1 [Reference]	NA	.01
Intermediate tertile	786	99	1.37 (0.99-1.89)	.06	
Highest tertile	788	117	1.55 (1.13-2.13)	.006	
Model 3					
Lowest tertile	465	37	1 [Reference]	NA	.005
Intermediate tertile	454	58	1.73 (1.10-2.70)	.02	
Highest tertile	453	68	1.98 (1.28-3.07)	.002	

Abbreviations: NA, not applicable; OR, odds ratio.

^a^ Model 1 is the unadjusted model. Model 2 is adjusted for characteristics of the child (sex, gestational age at birth), mother (age, education status, prepregnancy body mass index, passive smoking status, number of deliveries, mode of delivery), father (education status), and family (annual family income). Model 3 is model 2 plus blood lead levels in children.

^b^ Test for trend based on the variable containing the median value for each tertile.

**Figure 2.**
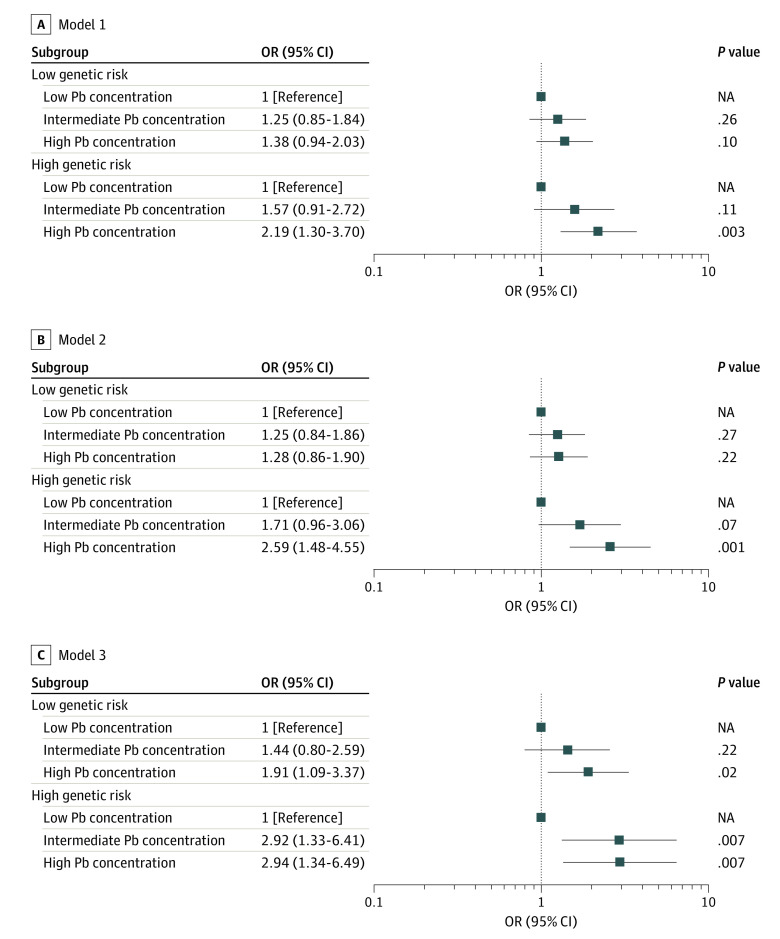
Risk for Cognitive Developmental Delay According to Prenatal Lead (Pb) Exposure and Genetic Categories of Children Odds ratios (ORs) for cognitive developmental delay were estimated according to Pb and genetic risk categories using logistic regression. Model 1 is the unadjusted model. Model 2 was adjusted for characteristics of the child (sex, gestational age at birth), mother (age, education status, prepregnancy body mass index, passive smoking status, number of deliveries, mode of delivery), father (education status), family (annual family income), and the top 10 principal components of ancestry and genotyping batch. Model 3 was adjusted for covariates in model 2 and blood Pb levels in children. NA indicates not applicable.

For genetic analysis of this study population, the quantile-quantile plots did not reveal any evidence of stratification in the population (inflation factor λ = 1.005) (eFigure 3 in Supplement 1), and the scree plot showed that the top 10 principal components can explain the main genetic stratification of the study population (eFigure 4 in Supplement 1). The 58 SNVs for PRS construction are shown in eTable 8 in Supplement 1. We observed that PRS was significantly associated with MDI (eTable 9 in Supplement 1), which implies that the cognitive PRS constructed in this study performed well. A significant joint association of prenatal Pb exposure and genetic factors for CDD risk was found. The ORs (95% CIs) for CDD risk in children with different genetic backgrounds according to tertiles of maternal plasma Pb concentrations are shown in Figure 2 (full regression analysis are provided in eTable 11 in Supplement 1). A significant association was found between CDD risk and increasing maternal plasma Pb concentrations among children with high genetic risk (highest vs lowest tertile: crude OR, 2.19; 95% CI, 1.30-3.70) (Figure 2A). After adjustment for demographic confounders, the adjusted OR of CDD risk associated with maternal plasma Pb concentrations was 2.59 (95% CI, 1.48-4.55) (highest vs lowest tertile) among children with high genetic risk (Figure 2B), and this phenomenon was also present in the model that was additionally adjusted for children’s blood Pb levels (adjusted OR, 2.94; 95% CI, 1.34-6.49) (Figure 2C).

By calculating the RERI and AP, we found a positive additive interaction between prenatal Pb exposure and genetic factors for CDD risk (Table 3). In particular, for children with a high genetic risk and a high tertile of maternal plasma Pb concentrations, the RERI was 0.84 (95% CI, 0.01-1.68) (adjusted for demographic confounding factors), indicating that there would be a 0.84 relative excess risk attributed to the additive interaction, accounting for 41% (95% CI, 7%-75%) of the risk of CDD in children exposed to both high maternal plasma Pb concentrations and high genetic risk.

**Table 3.** Interaction Between Highest Tertile Prenatal Lead Exposure and Genetic Categories

Model^a^	High genetic risk	
	RERI (95% CI)^b^	AP (95% CI)^b^
1	0.71 (−0.11 to 1.52)	0.35 (−0.01 to 0.71)
2	0.84 (0.01 to 1.68)	0.41 (0.07 to 0.75)
3	0.57 (−0.98 to 2.12)	0.21 (−0.31 to 0.72)

Abbreviations: AP, attributable proportion due to the interaction; RERI, relative excess risk due to the interaction.

^a^ Model 1 is the unadjusted model. Model 2 is adjusted for characteristics of the child (sex, gestational age at birth), mother (age, education status, prepregnancy body mass index, passive smoking status, number of deliveries, mode of delivery), father (education status), and family (annual family income). Model 3 is model 2 plus blood lead levels in children.

^b^ To estimate the RERI and AP, the low prenatal lead exposure and low genetic risk categories were reference categories.

## Discussion

This birth cohort study found that prenatal Pb exposure was associated with an elevated risk of CDD in children. The overall CDD incidence increased with both prenatal Pb exposure and genetic risk, with the greatest CDD risk observed among children with high prenatal Pb exposure and high genetic risk. Quantitative data regarding the additive interaction between prenatal Pb exposure and genetic factors associated with CDD showed a relative excess risk.

This study showed that even low levels of prenatal Pb exposure were significantly associated with children’s cognitive development at age 2 years, and neurocognitive toxicity of prenatal Pb exposure may be more critical than childhood exposure. Our findings are consistent with some studies^10,33^ but not others.^34,35^ The discrepancy among these studies might be related to the diversity in prenatal Pb exposure measurement and timing.^36^ As an epidemiologic investigation throughout pregnancy found, the association between Pb exposure and infant mental development is most pronounced in the first trimester (time for prenatal Pb measuring in our study).^36^ Regarding the mechanism, the brain matures most rapidly, and Pb penetrates the blood-brain barrier more easily during the fetal period, making fetal neurodevelopment more susceptible to Pb.^37^ In addition, our findings suggest that the neurocognitive toxicity of prenatal Pb exposure might be more prominent in girls. Sex differences in the association between Pb exposure and cognitive development have also been found in previous studies.^13,38^ Thus, the sex-specific association of Pb exposure with cognition is worth particular attention.

We found a relatively low maternal plasma concentration of Pb (GM, 7 ng/mL) in this study’s population. Higher maternal Pb exposure may exist elsewhere in China (eg, GM Pb concentrations of 45 ng/mL in Shanxi Province, China).^39^ Since hemolysis may affect plasma, making it challenging to measure Pb exposure well, relevant studies are still insufficient. To minimize measurement errors,^24^ several efforts were made in this study, such as using clean tubes free of metals, blank and quality control samples analyzed every 30 samples in all batches, and random selection of some samples for repeated measurements to ensure reliability and accuracy. In addition to being associated with environmental exposures in different geographic areas, plasma Pb concentrations in pregnant women could be associated with calcium supplementation inhibiting the movement of Pb from the bones and reducing the absorption of Pb from the diet.^8^ The GM of children’s blood Pb levels in our study (4.39 μg/dL; 2016-2019) was similar to those on a national scale in China (5.97 μg/dL; 2001-2018)^5^ and higher than those in France (1.49 μg/dL; 2008-2009)^40^ and the US (0.83 μg/dL; 2011-2016).^41^ This finding may be associated with emerging sources of Pb pollution, such as transport and e-waste, in China.^7^

Furthermore, we evaluated the neurocognitive toxicity of Pb based on different genetic backgrounds by introducing PRS for cognitive ability. We found that genetic factors might modify CDD risk caused by prenatal Pb exposure. Quantitative data on the additive interactions between prenatal Pb exposure and genetic factors revealed significant relative excess risk in the model adjusted for demographic confounders. However, the excess risk did not reached statistical significance in the models with no adjustment for confounders and additional adjustment for children’s blood Pb levels. This suggests that potential demographic confounders and childhood Pb exposure levels may be important factors when assessing the additive interaction between prenatal Pb exposure and genetics in children’s CDD risk. The results indicate that genetic background may play a role in altering the developing brain’s response to Pb and may be important for better understanding the molecular basis of neurobehavioral developmental delays due to childhood Pb poisoning. These findings are similar to some studies that examined the involvement of a single genetic variant in the association between Pb exposure and neurocognitive impairment. One clinical investigation identified increased neurologic harm to children’s neurodevelopment from the *GRIN2A* and *GRIN2B* genetic variants in combination with Pb exposure.^42^ Our findings support those of existing studies that genetic factors might interact with nongenetic exposures in neurocognitive development.

### Strengths and Limitations

This cohort study included a large number of mother-child pairs and a wide range of information on parental education, household income, mode of delivery, tests of children’s cognitive abilities, toxic heavy metal exposure, and genetic variations, which allowed us to prospectively explore the role of genetic factors in enhancing Pb neurocognitive toxicity. The novel findings on modifying Pb neurotoxicity by genetic background might prompt the development of more sensible prevention strategies for Pb exposure during pregnancy.

However, the limitations of this study should also be considered. First, home environment and parents’ cognitive ability, which may additionally interact with genetics and prenatal Pb exposure on CDD, were not measured, although we carefully adjusted for confounders such as gestational age at birth, education status of mothers and fathers, and annual family income, in the models. Second, Pb levels in cord blood and maternal whole blood samples, if measured, would have allowed for a better assessment of Pb neurocognitive toxicity. Finally, Pb concentrations of maternal blood were measured only once during the first trimester and might not reflect Pb exposure throughout pregnancy.

## Conclusions

In this cohort study, our findings indicate that prenatal exposure to even low levels of Pb was associated with increased CDD risk in children, especially in those with a high genetic risk. Prenatal Pb exposure and genetic background may jointly contribute to an increased risk of CDD for children. Additional studies are warranted to confirm our findings for improving children’s cognitive ability.

## Notes

Supplement 1. **eMethods 1.** Chinese Revision of Bayley Scale of Infant Development for Cognitive Assessment **eMethods 2.** Detailed Procedures for the Measurement of Pb Concentrations **eMethods 3.** DNA Extraction for Human Genome-Wide Arrays **eMethods 4.** Genotyping, Quality Control **eMethods 5.** The Imputation Process **eMethods 6.** Polygenic Risk Score (PRS) Calculation **eMethods 7.** Covariates **eTable 1.** Descriptive Characteristics of All Mother-Child Pairs **eTable 2.** Concentrations of Pb Among Study Participants **eTable 3.** Association Between CDD Risk and Prenatal Pb Exposure in Children With Different Genders **eTable 4.** Association Between CDD Risk and Pb Exposure in 2-Year-Old Children **eTable 5.** Sensitivity Analyses Restricted to Children With a Normal Range of Birth Weight **eTable 6.** Sensitivity Analysis Restricted to Pregnant Women Without Gestational Hypertension or Gestational Diabetes **eTable 7.** Associations Between MDD Risk and Prenatal Pb Exposure **eTable 8.** Summary Results of 58 SNPs Used for Polygenic Risk Score **eTable 9.** General Linear Model Evaluating Association Between PRS and MDI **eTable 10.** Full Regression Analysis Results for Table 2 **eTable 11.** Full Regression Analysis Results for Figure 2 **eFigure 1.** Flow Diagram of Mother-Child Pairs Exclusion **eFigure 2.** Association Between Baseline Status and Risk of CDD **eFigure 3.** Quantile-Quantile Plot and Genomic Inflation Factor Lambda for Associations With CDD Risk **eFigure 4.** Scree Plot of Eigenvalues for the Principal Component Analysis (PCA)
eReferences

Supplement 2.
Data Sharing Statement
