# Supplementary material for: Prenatal Lead Exposure, Genetic Factors, and Cognitive Developmental Delay
Source: JAMA Netw Open. 2023 Oct 23;6(10):e2339108. doi: 10.1001/jamanetworkopen.2023.39108 (PMC10594149; doi:10.1001/jamanetworkopen.2023.39108)
Supplement: Supplement 1. — eMethods 1. Chinese Revision of Bayley Scale of Infant Development for Cognitive Assessment eMethods 2. Detailed Procedures for the Measurement of Pb Concentrations eMethods 3. DNA Extraction for Human Genome-Wide Arrays eMethods 4. Genotyping, Quality Control eMethods 5. The Imputation Process eMethods 6. Polygenic Risk Score (PRS) Calculation eMethods 7. Covariates eTable 1. Descriptive Characteristics of All Mother-Child Pairs eTable 2. Concentrations of Pb Among Study Participants eTable 3. Association Between CDD Risk and Prenatal Pb Exposure in Children With Different Genders eTable 4. Association Between CDD Risk and Pb Exposure in 2-Year-Old Children eTable 5. Sensitivity Analyses Restricted to Children With a Normal Range of Birth Weight eTable 6. Sensitivity Analysis Restricted to Pregnant Women Without Gestational Hypertension or Gestational Diabetes eTable 7. Associations Between MDD Risk and Prenatal Pb Exposure eTable 8. Summary Results of 58 SNPs Used for Polygenic Risk Score eTable 9. General Linear Model Evaluating Association Between PRS and MDI eTable 10. Full Regression Analysis Results for Table 2 eTable 11. Full Regression Analysis Results for Figure 2 eFigure 1. Flow Diagram of Mother-Child Pairs Exclusion eFigure 2. Association Between Baseline Status and Risk of CDD eFigure 3. Quantile-Quantile Plot and Genomic Inflation Factor Lambda for Associations With CDD Risk eFigure 4. Scree Plot of Eigenvalues for the Principal Component Analysis (PCA) eReferences [file jamanetwopen-e2339108-s001.pdf]

Supplemental Online Content

Jia Z, Zhang H, Yu L, et al. Prenatal lead exposure, genetic factors, and cognitive developmental delay. JAMA Netw Open. 2023;6(10):e2339108. doi:10.1001/jamanetworkopen.2023.39108

eMethods 1. Chinese Revision of Bayley Scale of Infant Development for Cognitive Assessment

eMethods 2. Detailed Procedures for the Measurement of Pb Concentrations

eMethods 3. DNA Extraction for Human Genome-Wide Arrays

eMethods 4. Genotyping, Quality Control

eMethods 5. The Imputation Process

eMethods 6. Polygenic Risk Score (PRS) Calculation

eMethods 7. Covariates

eTable 1. Descriptive Characteristics of All Mother-Child Pairs

eTable 2. Concentrations of Pb Among Study Participants

eTable 3. Association Between CDD Risk and Prenatal Pb Exposure in Children With Different Genders

eTable 4. Association Between CDD Risk and Pb Exposure in 2-Year-Old Children

eTable 5. Sensitivity Analyses Restricted to Children With a Normal Range of Birth Weight

eTable 6. Sensitivity Analysis Restricted to Pregnant Women Without Gestational Hypertension or Gestational Diabetes

eTable 7. Associations Between MDD Risk and Prenatal Pb Exposure

eTable 8. Summary Results of 58 SNPs Used for Polygenic Risk Score

eTable 9. General Linear Model Evaluating Association Between PRS and MDI

eTable 10. Full Regression Analysis Results for Table 2

eTable 11. Full Regression Analysis Results for Figure 2

eFigure 1. Flow Diagram of Mother-Child Pairs Exclusion

eFigure 2. Association Between Baseline Status and Risk of CDD

eFigure 3. Quantile-Quantile Plot and Genomic Inflation Factor Lambda for Associations With CDD Risk

eFigure 4. Scree Plot of Eigenvalues for the Principal Component Analysis (PCA)

eReferences

This supplemental material has been provided by the authors to give readers additional information about their work.

© 2023 Jia Z et al. *JAMA Network Open.*

eMethods 1. Chinese Revision of Bayley Scale of Infant Development for Cognitive Assessment

Chinese revision of Baylcy Scale of Infant Development (BSID-CR) is a variant of the Bayley Scales of Infant Development (BSID-1)[1] which was adapted for application in the Chinese population.[2,3] As with its precursor, the BSID-CR includes scales for mental and psychomotor factors. The mental (psychological) and psychomotor development indices (MDI and PDI, respectively) were derived from the raw scores of the appropriate scales which were then adjusted based on the child's age as necessary. This was achieved via the formula: MDI or PDI = 100 + (X - Xm) / sd × 16, where Xm and sd are the mean and standard deviation of the abovementioned raw scores, respectively, and X is the raw score of the subject child.[3] Specifically, MDI is a representation of a child's cognitive development with respect to language ability, generalization, categorization, memory, and social skills among other aspects, while PDI serves the same purpose for psychomotor development, such as muscle coordination, and gross and fine manipulative skills. Overall, children with better cognitive development tend to have higher MDI scores, and for this study, the BSID-CR grading scale standard[3] was applied, whereupon an MDI $\leqslant$ 79 was considered as cognitive developmental delay (CDD). Children with PDI $\leqslant$ 79 was considered as motor developmental delay (MDD). Tests were administered by mental healthcare professionals who were not informed of the children's medical records (including family background, genetic factors, Pb exposure levels, etc.). For quality control, the entire evaluation process was monitored and fully documented, and the tests were conducted by similarly blind research assistants at the study hospital in a secluded area where background noise was at a minimum.

© 2023 Jia Z et al. *JAMA Network Open.*

eMethods 2. Detailed Procedures for the Measurement of Pb Concentrations

The plasma sample was properly mixed after being defrosted at 4°C, and a 90 µL aliquot was taken at room temperature (20-25°C) into a fresh tube for metal analysis. Samples were first diluted with ultrapure water (1:10) and then shaken to mix. The mixture was then 1:1 acidified with a mixture of Triton 0.04%, butanol 1%, and nitric acid 2% (1:1:1, v/v/v) and digested at room temperature overnight after vortex mixing. Personnel who weren't aware of the grouping information performed the assays. All participants' plasma samples were randomly tested and quantified. The method's accuracy was tested using certified reference materials (ClinChek® human plasma controls for trace elements no. 8883 and no. 8884, Recipe, Munich, Germany). The reference materials' metrics were all within the recommended range for Pb. Blank samples and quality control samples were analyzed every 30 samples in all batches. Inductively coupled plasma mass spectrometry (ICP-MS, Agilent 7900, Agilent Technologies, USA) was used to analyze the obtained solutions. Furthermore, to assess the whole blood Pb for children, the venous blood samples from 2-year-old children were measured using a Trace Element Analyzer AS-9000C (Wuhan Aoshou Medical Technology Co., Ltd) in accordance with standard operating procedures. For all Pb concentrations, the coefficients of variation (CVs) were less than 10% intra- and inter-day, while the limit of detection (LOD) for plasma Pb in mothers (using ICP-MS) was 0.005 ng/ml, and that of the blood Pb in children (using the trace element analyzer AS-9000C) was 0.001 µg/dL. None of the concentrations recorded in collected samples were lower than the respective LOD.

© 2023 Jia Z et al. *JAMA Network Open.*

eMethods 3. DNA Extraction for Human Genome-Wide Arrays

Upon collection at delivery, samples of cord blood were placed in EDTA-coated vacuum tubes, centrifuged, and placed at -80°C until detection. DNA of leukocytes in the samples were extracted and then evaluated (OD260/280 ratio and concentration) using a NanoDrop Spectrophotometers (Thermo Scientific, Wilmington, DE, USA). The DNA with a OD260/280 ratio of 1.7 - 2.0, a OD260/230 ratio of 2.0 - 2.2, and haven't been degraded was performed subsequent genome-wide arrays.

eMethods 4. Genotyping, Quality Control

Genotyping was carried out and completed, both per the manufacturer's protocols, using the Illumina Infinium Asian Screening Array v1.0 BeadChip (Illumina Inc., San Diego, CA, United States) (which contains ~ 700,000 markers) and Illumina iScan System, respectively. The prospective birth cohort in Wuhan, which consisted of 2361 children, was genotyped for the PRS application.

For the present study, standard quality control was applied at both the sample and variant levels similar to previously reported research. Genotyping quality control was performed using PLINK 1.9 (https://www.cog-genomics.org/plink/1.9/)[4]. We excluded samples with call rate <95% or sex discrepancy, or samples with extreme heterozygosity (> 6 SD from the mean). Identity by descent (IBD) analysis to detect cryptic relatedness and members with low call rates were excluded when PI_HAT > 0.185 for a pair of samples. Population structure was evaluated by principal component analysis (PCA) based on LDpruned ($r^2 < 0.8$)

© 2023 Jia Z et al. *JAMA Network Open.*

autosomal SNPs. We further excluded SNPs not in Hardy-Weinberg equilibrium (HWE, $P < 10^{-6}$). Additional SNPs with MAF < 0.01 and call rate < 95% were excluded.

Quantile-quantile (Q-Q) plots were generated to compare observed *P*-value distributions to expected distributions under a null hypothesis (no stratification of populations). In this study population, the Q-Q plots did not reveal any evidence of stratification in the population (inflation factor $\lambda = 1.005$) (eFigure 3 in Supplement 1).

eMethods 5. The Imputation Process

Genotype imputation is an effective and critical method for estimating unobserved genotypes in genomic data from single nucleotide polymorphism (SNP) genotyping arrays. Fine mapping of variants in genome-wide association studies (GWAS) can be greatly improved by the imputation of optimal reference panels based on population-specific haplotypes. Previous reference panel datasets with limited Chinese samples have limited the quality of reference panel imputation in current Chinese population studies. In addition, insufficient sequencing depth in the constructed datasets may undermine the ability to detect rare variants in imputation. Therefore, population-specific reference panels constructed from large, in-depth WGS datasets of Chinese populations are essential for accurate and comprehensive imputation of genotyping arrays and sequencing data in Chinese populations. The China Metabolic Analysis Project (ChinaMAP) has achieved high-quality imputation for a Chinese genomic dataset of 10,155 unlinked Chinese individuals with 59.01 M SNPs. ChinaMAP's reference samples provide more comprehensive imputation and more novel findings for genetic studies of the Chinese population. ChinaMAP's imputation server can be

© 2023 Jia Z et al. *JAMA Network Open.*

found at The ChinaMAP imputation server is available on the ChinaMAP website (www.mbiobank.com) for genotype imputation, downloading results, and variant databases. For the Infinium Asian Screening Array (ASA) imputation, the ChinaMAP Reference Board has demonstrated excellent performance in terms of the number of well-imputed variants and the quality of the shared imputed variants.[5]

Eligible samples and genotype data were phased with Eagle2 and imputed with ChinaMAP, using default parameters, and ChinaMAP dataset (Phase 1 Integrated Variant Set Release, spanning 10155 samples) was used as a reference. SNPs with MAF < 0.01 were excluded from the analysis.

eMethods 6. Polygenic Risk Score (PRS) Calculation

In the present study, we generated the PRS by multiplying the genotype dosage of each risk allele for each variant by its respective weight (β), summing all variants together into a PRS. According to the number of risk alleles, each SNP was recorded as 0, 1, or 2. The PRS was calculated using the equation: PRS = $\sum_{j=1}^{M} \beta_j \times SNP_j$, where M is the total number of SNPs, and β_j is per allele for cognitive ability associated with SNP_j. The β were taken from a meta-analysis of genome-wide association studies in the CHARGE consortium on cognitive function.[6]

eMethods 7. Covariates

The following data was extracted from participants' medical records: mother's age; gestational age; mode of delivery; gestational diabetes; gestational hypertension; gender of the baby; and birth weight of the baby. The heights and weights of mothers were also

© 2023 Jia Z et al. *JAMA Network Open.*

collected, from which their body mass index (BMI) before pregnancy were carefully calculated. Standardized in-person interviews with appropriately trained staff at the hospital provided additional information (in particular, the mother's education level and extent of passive smoking, the father's education level, and annual household income). Passive smoking was defined as prenatal exposure to cigarette smoke during pregnancy (whether from the father or other people who smoked at home or work). The 10 principal components (PCs) to be chosen to adjust for potential confounding effects of population genetic group stratification according to scree plot analysis (eFigure 4 in Supplement 1).[7] Additionally, the blood Pb level of children involved in the study, as measured using a trace element analyzer (AS-9000C, Wuhan Aoshou Medical Technology Co., Ltd.), was also included as a covariate in the models. Genetic risk and prenatal Pb exposure were also included as covariates in the models when analysing additive interaction effects.

© 2023 Jia Z et al. *JAMA Network Open.*

eTable 1. Descriptive Characteristics of All Mother-Child Pairs

Characteristics	Mother-child pairs, No. (%)			
	Total (n = 2361)	Normal (n = 2069)	CDD (n = 292)	*P* value[a]
**Birth weight, mean (SD), g**	3334.5 (421.3)	3329.2 (417.8)	3372.0 (444.3)	.10
**Birth weight group**				
< 2500	51 (2.2)	45 (2.2)	6 (2.1)	
2500-4000	2180 (92.3)	1915 (92.6)	265 (90.8)	.40
> 4000	130 (5.5)	109 (5.3)	21 (7.2)	
**Gestational hypertension**				
No	2329 (98.6)	2042 (98.7)	287 (98.3)	.57
Yes	32 (1.4)	27 (1.3)	5 (1.7)	
**Gestational diabetes**				
No	2157 (91.4)	1892 (91.4)	265 (90.8)	.69
Yes	204 (8.6)	177 (8.6)	27 (9.2)	

Abbreviations: SD, standard deviation; CDD, cognitive developmental delay.

[a] *P* value for the difference according to the chi-square test and unpaired two-samples t-test.

eTable 2. Concentrations of Pb Among Study Participants[a]

Children's Group		Mothers, GM(GSD)	*P* Value†	Children, GM(GSD)	*P* Value[b]
Gender	Boys	7.47(0.18)	.33	4.46(0.06)	.12
	Girls	7.16(0.17)		4.31(0.05)	
Genetic risk	Low	7.27(0.15)	.70	4.39(0.05)	.98
	High	7.43(0.21)		4.37(0.07)	

Abbreviations: Pb, Lead; GM, Geometric mean; GSD, geometric standard deviation.

[a] The unit is ng/mL (to convert ng/mL to g/L, multiply by 1 × 10-6)

[b] *P* value for differences between various genders or genetic risks using non-parametric tests.

eTable 3. Association Between CDD Risk and Prenatal Pb Exposure in Children With Different Genders

Model	Prenatal Pb exposure	Total / CDD	OR (95%CI)[a]	*P* value	*P* for trend[b]
boys	low tertile	415 / 55	Reference	Reference	.22
	Intermediate tertile	387 / 71	1.44 (0.97 - 2.12)	.07	
	High tertile	438 / 78	1.34 (0.92 - 1.96)	.13	
girls	low tertile	372 / 21	Reference	Reference	.008
	Intermediate tertile	399 / 28	1.24 (0.69 - 2.25)	.48	
	High tertile	350 / 39	2.04 (1.16 - 3.58)	.01	

Abbreviations: CDD, cognitive developmental delay; Pb, Lead; OR, odds ratio; CI, confidence interval.

[a] Adjusted for characteristics of the child (gender, gestational age at birth), mother (age, education status, pre-pregnancy body mass index, passive smoking status, number of deliveries, mode of delivery), father (education status), and family (annual family income).

[b] Test for trend based on the variable containing the median value for each tertile.

© 2023 Jia Z et al. *JAMA Network Open.*

eTable 4. Association Between CDD Risk and Pb Exposure in 2-Year-Old Children

Model	Prenatal Pb exposure	Total / CDD	OR (95%CI)	*P* value	*P* for trend[a]
	low tertile	465 / 37	Reference	Reference	
Model 1	Intermediate tertile	454 / 58	0.84 (0.6 - 1.170)	.30	.28
	High tertile	453 / 68	1.24 (0.91 - 1.68)	.17	
	low tertile	465 / 37	Reference	Reference	
Model 2	Intermediate tertile	454 / 58	0.82 (0.58 - 1.15)	.24	.31
	High tertile	453 / 68	1.24 (0.91 - 1.69)	.18	
	low tertile	465 / 37	Reference	Reference	
Model 3	Intermediate tertile	454 / 58	0.82 (0.58 - 1.15)	.24	.30
	High tertile	453 / 68	1.24 (0.91 - 1.69)	.17	

Abbreviations: CDD, cognitive developmental delay; Pb, Lead; OR, odds ratio; CI, confidence interval.

Model 1: Unadjusted model.

Model 2: Adjusted for characteristics of the child (gender, gestational age at birth), mother (age, education status, pre-pregnancy body mass index, passive smoking status, number of deliveries, mode of delivery), father (education status), and family (annual family income).

Model 3: Model 2 + Prenatal Pb exposure (Ln transformed).

[a] Test for trend based on the variable containing the median value for each tertile.

eTable 5. Sensitivity Analyses Restricted to Children With a Normal Range of Birth Weight

Model	Prenatal Pb exposure	Total / CDD	OR (95%CI)	*P* value	*P* for trend[a]
	low tertile	727 / 69	Reference	Reference	
Model 1	Intermediate tertile	727 / 94	1.42 (1.02 to 1.97)	.04	.01
	High tertile	726 / 102	1.56 (1.13 to 2.16)	.007	
	low tertile	727 / 69	Reference	Reference	
Model 2	Intermediate tertile	727 / 94	1.43 (1.02 - 2.00)	.04	.02
	High tertile	726 / 102	1.52 (1.09 - 2.12)	.01	
	low tertile	430 / 34	Reference	Reference	
Model 3	Intermediate tertile	429 / 54	1.67 (1.05 - 2.66)	.03	.01
	High tertile	414 / 59	1.86 (1.17 - 2.94)	.008	

Abbreviations: CDD, cognitive developmental delay; Pb, Lead; OR=odds ratio; CI, confidence interval.

Model 1: Unadjusted model.

Model 2: Adjusted for characteristics of the child (gender, gestational age at birth), mother (age, education status, pre-pregnancy body mass index, passive smoking status, number of deliveries, mode of delivery), father (education status), and family (annual family income).

Model 3: Model 2 + BLLs in children.

[a] Test for trend based on the variable containing the median value for each tertile.

© 2023 Jia Z et al. *JAMA Network Open.*

eTable 6. Sensitivity Analysis Restricted to Pregnant Women Without Gestational Hypertension or Gestational Diabetes

Model	Prenatal Pb exposure	Total / CDD	OR (95%CI)	*P* value	*P* for trend[a]
	low tertile	709 / 69	Reference	Reference	
Model 1	Intermediate tertile	710 / 86	1.28 (0.91 - 1.79)	.15	.004
	High tertile	710 / 105	1.61 (1.16 - 2.22)	.004	
	low tertile	709 / 69	Reference	Reference	
Model 2	Intermediate tertile	710 / 86	1.27 (0.90 - 1.78)	.17	.01
	High tertile	710 / 105	1.55 (1.11 - 2.16)	.01	
	low tertile	422 / 37	Reference	Reference	
Model 3	Intermediate tertile	421 / 50	1.38 (0.87 - 2.19)	.18	.008
	High tertile	398 / 62	1.84 (1.18 - 2.88)	.008	

Abbreviations: CDD, cognitive developmental delay; Pb, Lead; OR=odds ratio; CI, confidence interval.
Model 1: Unadjusted model.
Model 2: Adjusted for characteristics of the child (gender, gestational age at birth), mother (age, education status, pre-pregnancy body mass index, passive smoking status, number of deliveries, mode of delivery), father (education status), and family (annual family income).
Model 3: Model 2 + BLLs in children.
[a] Test for trend based on the variable containing the median value for each tertile.

eTable 7. Associations Between MDD Risk and Prenatal Pb Exposure

Model	Prenatal Pb exposure	Total / MDD	OR (95%CI)	*P* value	*P* for trend[a]
	low tertile	787 / 50	Reference	Reference	
Model 1	Intermediate tertile	786 / 48	0.96 (0.64 - 1.44)	.84	.49
	High tertile	788 / 56	1.13 (0.76 - 1.67)	.55	
	low tertile	787 / 50	Reference	Reference	
Model 2	Intermediate tertile	786 / 48	0.97 (0.64 - 1.46)	.87	.54
	High tertile	788 / 56	1.12 (0.75 - 1.67)	.60	
	low tertile	465 / 16	Reference	Reference	
Model 3	Intermediate tertile	454 / 16	1.03 (0.50 - 2.11)	.94	.23
	High tertile	453 / 24	1.44 (0.74 - 2.80)	.28	

Abbreviations: MDD, motor developmental delay; Pb, Lead; OR, odds ratio; CI, confidence interval.
Model 1: Unadjusted model.
Model 2: Adjusted for characteristics of the child (gender, gestational age at birth), mother (age, education status, pre-pregnancy body mass index, passive smoking status, number of deliveries, mode of delivery), father (education status), and family (annual family income).
Model 3: Model 2 + blood Pb levels in children.
[a] Test for trend based on the variable containing the median value for each tertile.

© 2023 Jia Z et al. *JAMA Network Open.*

eTable 8. Summary Results of 58 SNPs USED for Polygenic Risk Score

NO.	MarkerName	Chr. (hg38)	Position (hg38)	A1	A2	Beta
1	rs7713692	5	2143361	t	c	0.1146
2	rs7379133	5	2149096	a	t	0.1113
3	rs4763555	12	10568857	a	g	0.0283
4	rs4541094	16	15776878	t	c	0.0311
5	rs2842385	6	19078043	a	g	-0.0311
6	rs9477970	6	19082432	a	t	0.0332
7	rs1480997	8	21626946	a	g	0.0558
8	rs17014925	3	24606037	a	g	-0.0621
9	rs2347503	8	32375311	a	g	-0.0465
10	rs2239647	14	32823537	a	c	-0.0266
11	rs2143975	14	32828192	c	g	-0.028
12	rs10145461	14	32829124	t	g	0.0256
13	rs4261436	14	32830276	t	c	0.0263
14	rs17522122	14	32833676	t	g	-0.0311
15	rs12885467	14	32834582	t	c	0.0306
16	rs1523041	3	35495841	c	g	0.0258
17	rs2836923	21	39152310	t	c	-0.028
18	rs2150415	21	39243441	t	c	-0.0277
19	rs2836974	21	39291255	a	g	0.0281
20	rs2735306	21	39341208	a	g	0.0258
21	rs307120	18	40039975	t	c	-0.0277
22	rs1840593	21	42206563	a	g	-0.0293
23	rs134902	22	42287991	a	g	-0.0269
24	rs80506	22	42293134	a	t	-0.0275
25	rs157582	19	44892962	t	c	-0.0355
26	rs10119	19	44903416	a	g	-0.0446
27	rs2289681	17	44911720	t	c	0.038
28	rs4420638	19	44919689	a	g	0.0459
29	rs11719291	3	48698273	a	g	-0.04
30	rs6997	3	49416401	t	c	0.0267
31	rs4283605	3	49641218	a	g	-0.0296
32	rs4282054	3	52532049	t	c	-0.0255
33	rs773107	12	55975722	a	g	-0.0267
34	rs773114	12	55985276	a	t	-0.0258
35	rs877636	12	56086799	a	g	-0.0313
36	rs7997462	13	57683031	t	c	0.0299
37	rs6561937	13	57683533	a	t	0.0321
38	rs9537821	13	57828637	a	g	0.0291
39	rs302874	17	58690635	a	g	-0.0293

© 2023 Jia Z et al. *JAMA Network Open.*

eTable 8. Summary Results of 58 SNPs used for Polygenic Risk Score (continued)

NO.	MarkerName	Chr. (hg38)	Position (hg38)	A1	A2	Beta
40	rs10853008	17	58916046	t	c	0.0302
41	rs7208505	17	59110368	a	g	0.0257
42	rs1942771	18	73544510	a	c	0.0295
43	rs291482	3	73762418	a	g	0.0285
44	rs13034462	2	79892368	t	g	-0.0603
45	rs12987105	2	79910411	t	c	-0.0605
46	rs12658240	5	81877446	t	c	0.0291
47	rs17518584	3	85555773	t	c	0.0282
48	rs6452788	5	88417096	a	g	0.0288
49	rs12091709	1	89878392	a	g	-0.1154
50	rs1933720	6	97849956	t	c	-0.0292
51	rs1933721	6	97865347	c	g	-0.0268
52	rs4339469	6	97921354	t	g	0.027
53	rs9375195	6	98114844	a	g	0.0329
54	rs10457441	6	98124244	t	c	-0.0324
55	rs6838310	4	117956978	t	c	-0.029
56	rs2303319	2	161368153	t	c	0.0451
57	rs9814607	3	171967792	a	t	0.0413
58	rs1920975	4	177556298	a	g	-0.0254

eTable 9. General Linear Model Evaluating Association between PRS and MDI

Model	N	β (95% CI)	*P* value
Crude	2361	4.19 (0.37 to 8.00)	.03
Adjusted†	2361	3.08 (-0.66 to 6.81)	.11

Abbreviations: Pb, Lead; OR=odds ratio; CI, confidence interval.

† Adjusted for characteristics of the child (gender, gestational age at birth), mother (age, education status, pre-pregnancy body mass index, passive smoking status, number of deliveries, mode of delivery), father (education status), family (annual family income), and the top 10 principal components of ancestry and genotyping batch.

© 2023 Jia Z et al. *JAMA Network Open.*

eTable 10. Full Regression Analysis Results for Table 2

Model	Variables		OR (95%CI)	*P* value
Model 1	Prenatal Pb exposure	Intermediate tertile vs. low tertile	1.35 (0.98 - 1.85)	.06
		High tertile vs. low tertile	1.63 (1.20 - 2.22)	.002
Model 2	Prenatal Pb exposure	Intermediate tertile vs. low tertile	1.37 (0.99 - 1.89)	.06
		High tertile vs. low tertile	1.55 (1.13 - 2.13)	.006
	Maternal age at delivery (continous)		1.01 (0.97 - 1.05)	.74
	pre-pregnancy BMI (continous)		1.04 (0.99 - 1.08)	.11
	Child gender	Girls vs. Boys	0.43 (0.33 - 0.56)	< .001
	Parity	≥ 2 vs. 1	0.99 (0.69 - 1.44)	.97
	Annual family income, yuan/y	50,000-99,000 vs. ≥ 100,000	1.06 (0.80 - 1.42)	.68
		< 50,000 vs. ≥ 100,000	1.64 (1.13 - 2.37)	.009
	Mode of delivery	Cesarean section vs. Vaginal birth	1.23 (0.95 - 1.60)	.11
	Paternal education status, y	Some college vs. ≥ college	0.94 (0.69 - 1.28)	.69
		≤ High school vs. ≥ college	1.46 (1.05 - 2.03)	.02
	Maternal education status, y	Some college vs. ≥ college	1.12 (0.83 - 1.52)	.46
		≤ High school vs. ≥ college	1.23 (0.87 - 1.75)	.25
	Gestational age group at birth, wk	≤ 37 vs. > 37	0.69 (0.31 - 1.54)	.37
	Passive smoking during pregnancy	Yes vs.No	0.79 (0.59 - 1.07)	.13

© 2023 Jia Z et al. *JAMA Network Open.*

eTable 10. Full Regression Analysis Results for Table 2 (continued)

Model	Variables		OR (95%CI)	*P* value
Model 3	Prenatal Pb exposure	Intermediate tertile vs. low tertile	1.73 (1.10 - 2.70)	.02
		High tertile vs. low tertile	1.98 (1.28 - 3.07)	.002
	blood Pb levels in children (Ln transformed)		1.26 (0.80 - 1.97)	.32
	Maternal age at delivery, mean (SD), y		0.99 (0.94 - 1.05)	.83
	Pre-pregnancy BMI, mean (SD), kg/m2		0.99 (0.93 - 1.05)	.78
	Child gender	Girls vs. Boys	0.33 (0.23 - 0.48)	< .001
	Parity	≥ 2 vs. 1	1.13 (0.69 - 1.87)	.62
	Annual family income, yuan/y	50,000-99,000 vs. ≥ 100,000	0.96 (0.65 - 1.41)	.82
		< 50,000 vs. ≥ 100,000	1.33 (0.79 - 2.24)	.29
	Mode of delivery	Cesarean section vs. Vaginal birth	1.14 (0.80 - 1.62)	.46
	Paternal education status, y	Some college vs. ≥ college	0.93 (0.61 - 1.42)	.74
		≤ High school vs. ≥ college	1.54 (0.99 - 2.39)	.05
	Maternal education status, y	Some college vs. ≥ college	1.31 (0.87 - 1.95)	.20
		≤ High school vs. ≥ college	1.30 (0.81 - 2.08)	.29
	Gestational age group at birth, wk	≤ 37 vs. > 37	1.17 (0.39 - 3.53)	.78
	Passive smoking during pregnancy	Yes vs.No	0.74 (0.49 - 1.13)	.17

Abbreviations: Pb, Lead; OR, odds ratio; CI, confidence interval.

Model 1: Unadjusted model.

Model 2: Adjusted for characteristics of the child (gender, gestational age at birth), mother (age, education status, pre-pregnancy body mass index, passive smoking status, number of deliveries, mode of delivery), father (education status), and family (annual family income).

Model 3: Model 2 + blood Pb levels in children.

© 2023 Jia Z et al. *JAMA Network Open.*

eTable 11. Full Regression Analysis Results for Figure 2

Model	Variables		OR (95%CI)	*P* value
Model 1	low genetic risk	Intermediate Pb exposure vs. low Pb exposure	1.25 (0.85 - 1.84)	.26
		High Pb exposure vs. low Pb exposure	1.38 (0.94 - 2.03)	.10
	High genetic risk	Intermediate Pb exposure vs. low Pb exposure	1.57 (0.91 - 2.72)	.11
		High Pb exposure vs. low Pb exposure	2.19 (1.30 - 3.7)	.003
Model 2	low genetic risk	Intermediate Pb exposure vs. low Pb exposure	1.25 (0.84 - 1.86)	.27
		High Pb exposure vs. low Pb exposure	1.28 (0.86 - 1.9)	.22
	Maternal age at delivery (continous)		0.99 (0.94 - 1.04)	.60
	pre-pregnancy BMI (continous)		1.05 (1.00 - 1.11)	.07
	Child gender	Girls vs. Boys	0.47 (0.33 - 0.65)	< .001
	Parity	≥ 2 vs. 1	0.91 (0.56 - 1.49)	.72
	Annual family income, yuan/y	50,000-99,000 vs. ≥ 100,000	1.22 (0.85 - 1.75)	.28
		< 50,000 vs. ≥ 100,000	1.92 (1.22 - 3.03)	.005
	Mode of delivery	Cesarean section vs. Vaginal birth	1.22 (0.88 - 1.68)	.23
	Paternal education status, y	Some college vs. ≥ college	1.14 (0.77 - 1.67)	.51
		≤ High school vs. ≥ college	1.69 (1.11 - 2.58)	.01
	Maternal education status, y	Some college vs. ≥ college	1.12 (0.77 - 1.63)	.54
		≤ High school vs. ≥ college	0.88 (0.56 - 1.4)	.60
	Gestational age group at birth, wk	≤ 37 vs. > 37	0.41 (0.12 - 1.34)	.14
	Passive smoking during pregnancy	Yes vs.No	0.88 (0.61 - 1.28)	.51
	Principal component 1		13.39 (0.02 - 11341.26)	.45
	Principal component 2		0.87 (0 - 1252.55)	.97
	Principal component 3		1.07 (0 - 3235.36)	.99
	Principal component 4		2.77 (0 - 6520.55)	.80
	Principal component 5		0.09 (0 - 193.06)	.54

© 2023 Jia Z et al. *JAMA Network Open.*

eTable 11. Full Regression Analysis Results for Figure 2 (continued)

Model	Variables		OR (95%CI)	*P* value
	Principal component 6		1.27 (0 - 2602.26)	.95
	Principal component 7		0.00 (0.00 - 7.41)	.15
	Principal component 8		1.77 (0 - 2828.59)	.88
	Principal component 9		0.32 (0 - 620.44)	.77
	Principal component 10		3.78 (0 - 7700.23)	.73
	High genetic risk	Intermediate Pb exposure vs. low Pb exposure	1.71 (0.96 - 3.06)	.07
		High Pb exposure vs. low Pb exposure	2.59 (1.48 - 4.55)	.001
	Maternal age at delivery (continous)		1.04 (0.98 - 1.11)	.21
	pre-pregnancy BMI (continous)		1.01 (0.93 - 1.09)	.87
	Child gender	Girls vs. Boys	0.32 (0.2 - 0.52)	<.001
	Parity	≥ 2 vs. 1	1.02 (0.55 - 1.88)	.96
Model 2	Annual family income, yuan/y	50,000-99,000 vs. ≥ 100,000	0.8 (0.49 - 1.3)	.36
		< 50,000 vs. ≥ 100,000	1.08 (0.54 - 2.16)	.83
	Mode of delivery	Cesarean section vs. Vaginal birth	1.3 (0.83 - 2.05)	.26
	Paternal education status, y	Some college vs. ≥ college	0.62 (0.36 - 1.07)	.09
		≤ High school vs. ≥ college	1.13 (0.65 - 1.98)	.66
	Maternal education status, y	Some college vs. ≥ college	1.02 (0.59 - 1.76)	.94
		≤ High school vs. ≥ college	2.09 (1.16 - 3.77)	.01
	Gestational age group at birth, wk	≤ 37 vs. > 37	1.5 (0.45 - 5.02)	.51
	Passive smoking during pregnancy	Yes vs.No	0.63 (0.37 - 1.07)	.09
	Principal component 1		0.00 (0.00 - 257.72)	.18
	Principal component 2		0.02 (0 - 28000.34)	.59
	Principal component 3		8531.18 (0.22 - 323554201.53)	.09
	Principal component 4		0.02 (0 - 2251.53)	.52

© 2023 Jia Z et al. *JAMA Network Open.*

eTable 11. Full Regression Analysis Results for Figure 2 (continued)

Model	Variables		OR (95%CI)	*P* value
Model 2	Principal component 5		17.09 (0 - 777483.69)	.60
	Principal component 6		29.48 (0 - 908671.75)	.52
	Principal component 7		0.03 (0 - 959.15)	.50
	Principal component 8		0.04 (0 - 2191.12)	.55
	Principal component 9		0.00 (0.00 - 18.78)	.16
	Principal component 10		0.2 (0.00 - 14804.78)	.78
Model 3	low genetic risk	Intermediate Pb exposure vs. low Pb exposure	1.44 (0.8 - 2.59)	.22
		High Pb exposure vs. low Pb exposure	1.91 (1.09 - 3.37)	.02
	blood Pb levels in children (Ln transformed)		1.21 (0.69 - 2.12)	.51
	Maternal age at delivery (continous)		0.94 (0.87 - 1.02)	.12
	pre-pregnancy BMI (continous)		1.01 (0.93 - 1.1)	.77
	Child gender	Girls vs. Boys	0.36 (0.22 - 0.57)	<.001
	Parity	≥ 2 vs. 1	1.34 (0.67 - 2.68)	.41
	Annual family income, yuan/y	50,000-99,000 vs. ≥ 100,000	1.2 (0.72 - 2)	.48
		< 50,000 vs. ≥ 100,000	1.5 (0.78 - 2.88)	.23
	Mode of delivery	Cesarean section vs. Vaginal birth	1.17 (0.74 - 1.84)	.50
	Paternal education status, y	Some college vs. ≥ college	1.15 (0.66 - 2.02)	.62
		≤ High school vs. ≥ college	2.07 (1.17 - 3.68)	.01
	Maternal education status, y	Some college vs. ≥ college	1.32 (0.79 - 2.22)	.28
		≤ High school vs. ≥ college	0.87 (0.45 - 1.67)	.67
	Gestational age group at birth, wk	≤ 37 vs. > 37	0.34 (0.04 - 2.71)	.31
	Passive smoking during pregnancy	Yes vs.No	0.93 (0.55 - 1.56)	.77
	Principal component 1		0.08 (0 - 21341.28)	.7
	Principal component 2		1.52 (0 - 124848)	.94

© 2023 Jia Z et al. *JAMA Network Open.*

eTable 11. Full Regression Analysis Results for Figure 2 (continued)

Model	Variables		OR (95%CI)	*P* value
Model 3	Principal component 3		1619.03 (0.02 - 117909074.21)	.20
	Principal component 4		21097.43 (0.38 - 1156701862.8)	.07
	Principal component 5		96.28 (0 - 3722482.73)	.40
	Principal component 6		36.56 (0 - 1744546.26)	.51
	Principal component 7		0.00 (0.00 - 2.8)	.08
	Principal component 8		56.61 (0 - 2834252.11)	.46
	Principal component 9		0.15 (0 - 7002.1)	.73
	Principal component 10		1.12 (0 - 53245.28)	.98
	High genetic risk	Intermediate Pb exposure vs. low Pb exposure	2.92 (1.33 - 6.41)	.007
		High Pb exposure vs. low Pb exposure	2.94 (1.34 - 6.49)	.007
	blood Pb levels in children (Ln transformed)		1.59 (0.63 - 4)	.33
	Maternal age at delivery (continous)		1.09 (1 - 1.18)	.05
	pre-pregnancy BMI (continous)		0.98 (0.88 - 1.09)	.72
	Child gender	Girls vs. Boys	0.22 (0.11 - 0.42)	<.001
	Parity	≥ 2 vs. 1	0.93 (0.4 - 2.14)	.86
	Annual family income, yuan/y	50,000-99,000 vs. ≥ 100,000	0.63 (0.33 - 1.2)	.16
		< 50,000 vs. ≥ 100,000	0.93 (0.34 - 2.56)	.89
	Mode of delivery	Cesarean section vs. Vaginal birth	1.26 (0.68 - 2.33)	.46
	Paternal education status, y	Some college vs. ≥ college	0.56 (0.28 - 1.14)	.11
		≤ High school vs. ≥ college	1 (0.47 - 2.12)	> .99
	Maternal education status, y	Some college vs. ≥ college	1.06 (0.52 - 2.13)	.88
		≤ High school vs. ≥ college	2.16 (1 - 4.68)	.05
	Gestational age group at birth, wk	≤ 37 vs. > 37	8.34 (1.49 - 46.83)	.02
	Passive smoking during pregnancy	Yes vs.No	0.48 (0.22 - 1.05)	.07

© 2023 Jia Z et al. *JAMA Network Open.*

eTable 11. Full Regression Analysis Results for Figure 2 (continued)

Model	Variables	OR (95%CI)	*P* value
	Principal component 1	0.00 (0.00 - 3857.22)	.30
	Principal component 2	0.02 (0.00 - 67108.52)	.62
	Principal component 3	67214.6 (0.08 - 56495342572.03)	.11
	Principal component 4	25.41 (0.00 - 33664006.6)	.65
	Principal component 5	31117.21 (0.03 - 29572700510.9)	.14
	Principal component 6	1105.06 (0 - 1244127275.6)	.32
	Principal component 7	3.03 (0.00 - 4826233.8)	.88
	Principal component 8	11.79 (0.00 - 13079381.58)	.73
	Principal component 9	2.37 (0.00 - 2741596.79)	.90
	Principal component 10	7.23 (0.00 - 10020416.63)	.78

Abbreviations: Pb, Lead; OR, odds ratio; CI, confidence interval.

Model 1: Unadjusted model.

Model 2: Adjusted for characteristics of the child (gender, gestational age at birth), mother (age, education status, pre-pregnancy body mass index, passive smoking status, number of deliveries, mode of delivery), father (education status), and family (annual family income).

Model 3: Model 2 + blood Pb levels in children.

© 2023 Jia Z et al. *JAMA Network Open.*

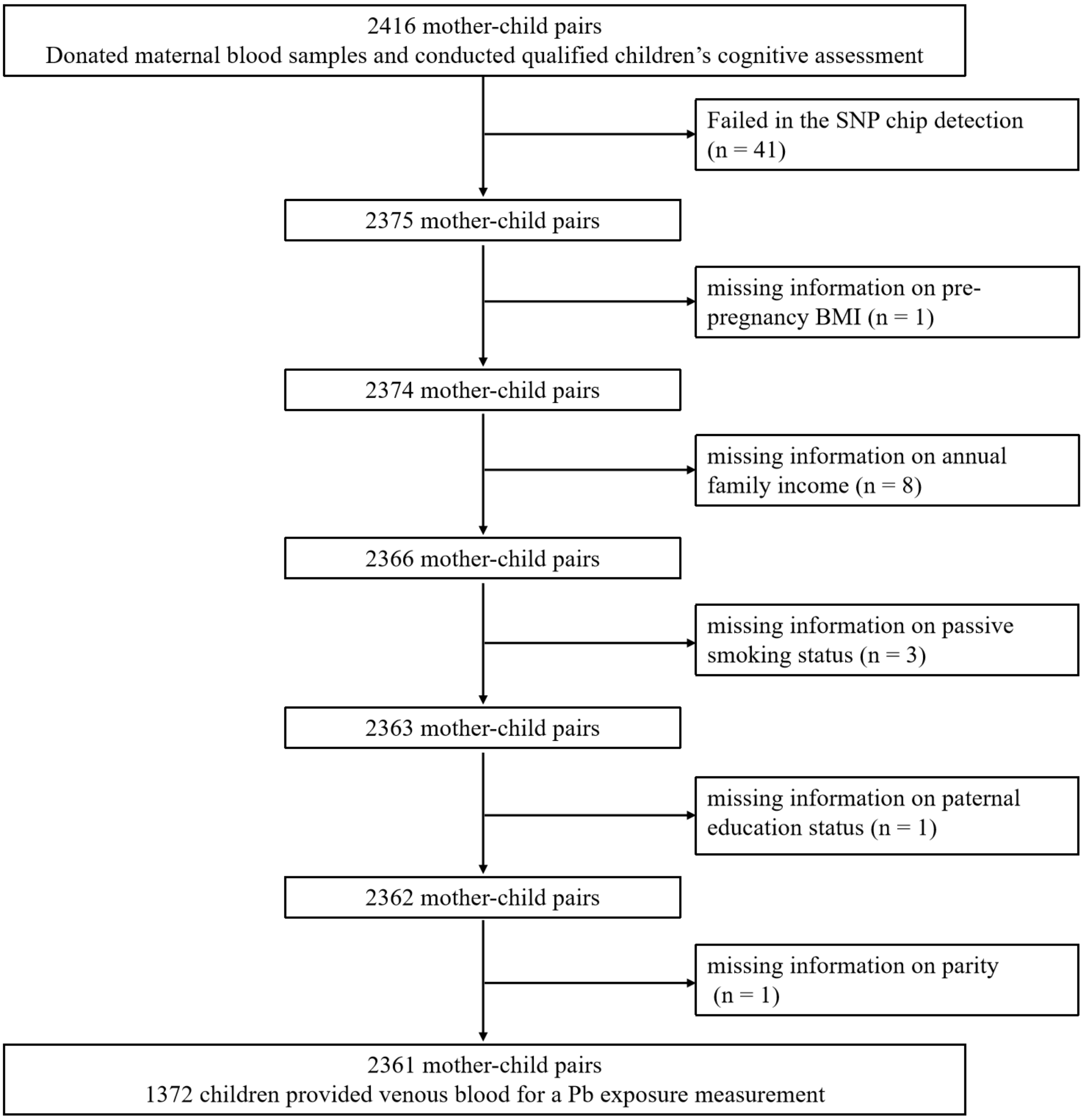

eFigure 1. Flow Diagram of Mother-Child Pairs Exclusion. This cohort study initially included 2416 mother-child pairs recruited from the birth cohort who had available maternal blood samples and qualified cognitive assessment for children. Among them, 41 participants whose blood samples failed in the SNP chip detection were excluded. Furthermore, participants with missing information on pre-pregnancy BMI (n = 1), annual family income (n = 8), passive smoking status (n = 3), paternal education status (n = 1), and parity (n = 1) were excluded. For these reasons, our final study population consisted of 2361 mother-child pairs. Furthermore, during the follow-up, 1372 children provided venous blood for a Pb exposure measurement at the study hospital at approximately 2 years old.

© 2023 Jia Z et al. *JAMA Network Open.*

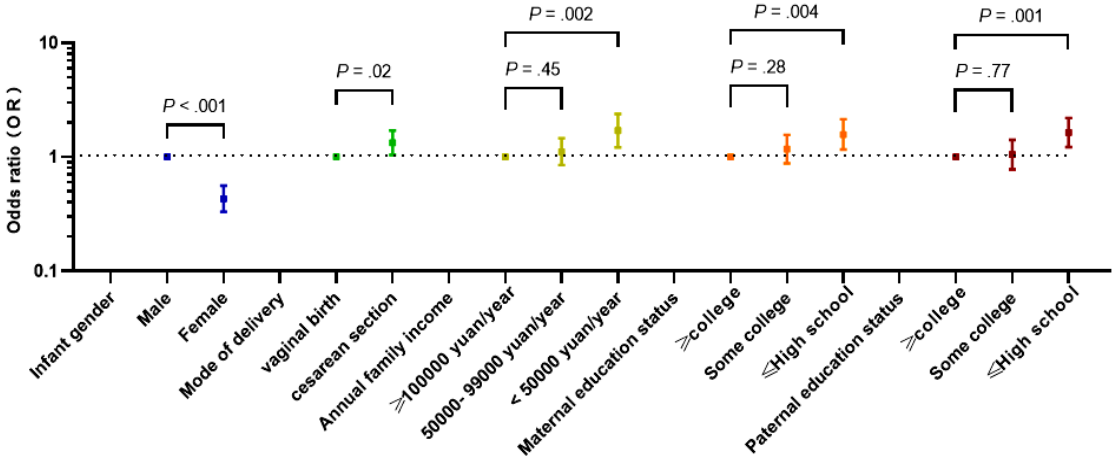

eFigure 2. Association Between Baseline Status and Risk of CDD. The OR (95% CI) for CDD risk at baseline status by child gender, mode of delivery, annual family income, maternal education, and paternal education. OR, odds ratio; CI, confidence interval.

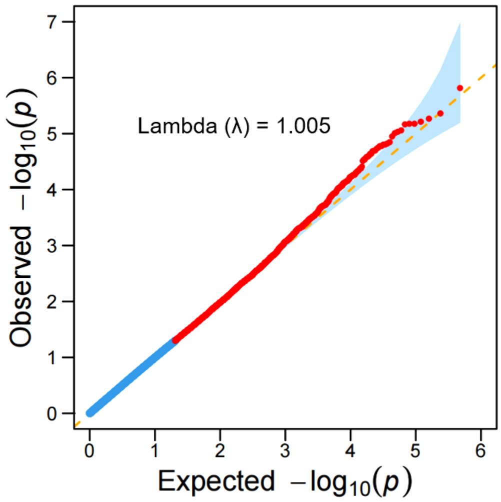

eFigure 3. Quantile-Quantile Plot and Genomic Inflation Factor Lambda for Associations With CDD Risk. The results were based on 2361 children in present study. The red dot represents the distribution of *P* values for the association. The observed versus expected χ^2 test statistics show no evidence for inflation of χ^2 tests (inflation factor $\lambda = 1.005$). No evidence of population stratification was observed in 2361 children. Adjusted for characteristics of the child (gender, gestational age at birth), mother (age, education status, pre-pregnancy body mass index, passive smoking status, number of deliveries, mode of delivery), father (education status), family (annual family income), and the top 10 principal components of ancestry and genotyping batch.

© 2023 Jia Z et al. *JAMA Network Open.*

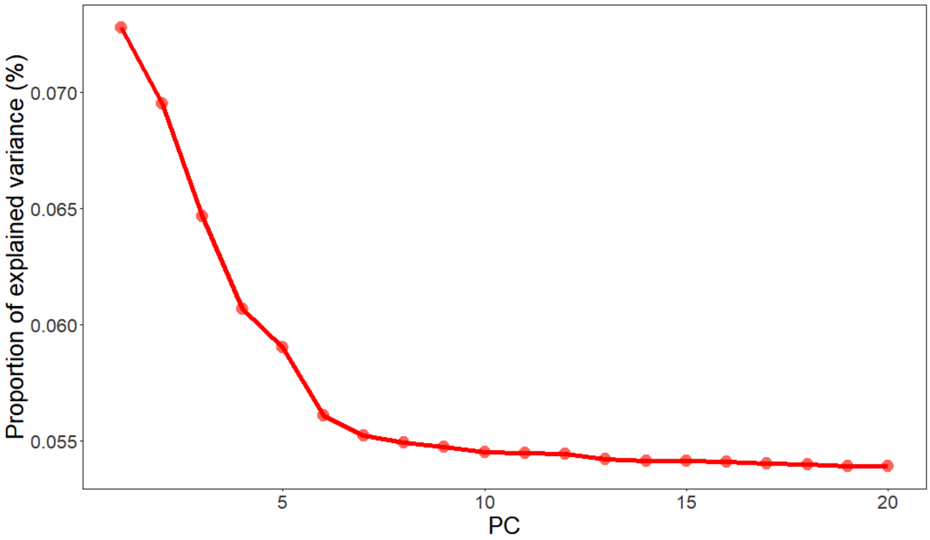

eFigure 4. Scree Plot of Eigenvalues for the Principal Component Analysis (PCA). The results are based on 2361 children in present study and the top 20 principal components were shown.

eReferences

1. Bayley N RG. Bayley scales of infant development. *Psychological Corporation New York.* 1969.
2. Huang H TS, Zhang YW. Standarization of Bayley scales of infant development in Shanghai. *Chin J Child Health.* 1993;1(3):158-160.
3. Yi SR, Luo XR, Yang ZW, Wan GB. The revising of the Bayley Scales of Infant Development (BSID) in China. *Chin J Clin Psychol.* 1993;1(2):71-75.
4. Thomason ME, Hect JL, Rauh VA, et al. Prenatal lead exposure impacts cross-hemispheric and long-range connectivity in the human fetal brain. *Neuroimage.* 2019;191:186-192. doi:10.1016/j.neuroimage.2019.02.017
5. Li L, Huang P, Sun X, et al. The ChinaMAP reference panel for the accurate genotype imputation in Chinese populations. *Cell Res.* 2021;31(12):1308-1310. doi:10.1038/s41422-021-00564-z
6. Davies G, Armstrong N, Bis JC, et al. Genetic contributions to variation in general cognitive function: a meta-analysis of genome-wide association studies in the CHARGE consortium (N=53949). *Mol Psychiatry.* 2015;20(2):183-192. doi:10.1038/mp.2014.188
7. Cattell RB. The Scree Test For The Number Of Factors. *Multivariate Behav Res.* 1966;1(2):245-276. doi:10.1207/s15327906mbr0102_10

© 2023 Jia Z et al. *JAMA Network Open.*
