# Supplementary material for: Prenatal Lead Exposure, Genetic Factors, and Cognitive Developmental Delay
Source: JAMA Netw Open. 2023 Oct 23;6(10):e2339108. doi: 10.1001/jamanetworkopen.2023.39108 (PMC10594149; doi:10.1001/jamanetworkopen.2023.39108)
Supplement: Supplement 2. — Data Sharing Statement [file jamanetwopen-e2339108-s002.pdf]

Data Sharing Statement

Jia. Prenatal Lead Exposure, Genetic Factors, and Cognitive Developmental Delay. *JAMA Netw Open*. Published October 23, 2023. doi:10.1001/jamanetworkopen.2023.39108

Data
Data available: No

Additional Information
Explanation for why data not available: Data will be made available on request.
